# Over 60 h of Stable Water‐Operation for N‐Type Organic Electrochemical Transistors with Fast Response and Ambipolarity

**DOI:** 10.1002/advs.202400872

**Published:** 2024-05-29

**Authors:** Tao Pan, Xinnian Jiang, Eveline R. W. van Doremaele, Junyu Li, Tom P. A. van der Pol, Chenshuai Yan, Gang Ye, Jian Liu, Wenjing Hong, Ryan C. Chiechi, Yoeri van de Burgt, Yanxi Zhang

**Affiliations:** ^1^ The Institute of Flexible Electronics (IFE, Future Technologies) & IKKEM & State Key Laboratory of Physical Chemistry of Solid Surfaces, College of Chemistry and Chemical Engineering Xiamen University Xiamen 361005 P. R. China; ^2^ Microsystems Department of Mechanical Engineering & Institute for Complex Molecular Systems Eindhoven University of Technology Eindhoven 5600 MB The Netherlands; ^3^ Sinopec Shanghai Research Institute of Petrochemical Technology Shanghai 201028 P. R. China; ^4^ Molecular Materials and Nanosystems & Institute for Complex Molecular Systems Eindhoven University of Technology Eindhoven 5600 MB The Netherlands; ^5^ Key Laboratory for the Green Preparation and Application of Functional Materials Hubei Key Laboratory of Polymer Materials School of Materials Science and Engineering Hubei University Youyi Road 368 Wuhan 430062 P. R. China; ^6^ State Key Laboratory of Polymer Physics and Chemistry Changchun Institute of Applied Chemistry Chinese Academy of Sciences Changchun Jilin 130022 P. R. China; ^7^ Department of Chemistry & Organic and Carbon Electronics Cluster North Carolina State University Raleigh NC 27695‐8204 USA

**Keywords:** ambipolar organic mixed ionic‐electronic conductors, organic electrochemical transistors, stability, threshold voltage

## Abstract

Organic electrochemical transistors (OECTs) are of great interest in low‐power bioelectronics and neuromorphic computing, as they utilize organic mixed ionic‐electronic conductors (OMIECs) to transduce ionic signals into electrical signals. However, the poor environmental stability of OMIEC materials significantly restricts the practical application of OECTs. Therefore, the non‐fused planar naphthalenediimide (NDI)‐dialkoxybithiazole (2Tz) copolymers are fine‐tuned through varying ethylene glycol (EG) side chain lengths from tri(ethylene glycol) to hexa(ethylene glycol) (namely P‐XO, X = 3–6) to achieve OECTs with high‐stability and low threshold voltage. As a result, the NDI‐2Tz copolymers exhibit ambipolarity, rapid response (<10 ms), and ultra‐high n‐type stability. Notably, the P‐6O copolymers display a threshold voltage as low as 0.27 V. They can operate in n‐type mode in an aqueous solution for over 60 h, maintaining an on‐off ratio of over 10^5^. This work sheds light on the design of exceptional n‐type/ambipolar materials for OECTs. It demonstrates the potential of incorporating these ambipolar polymers into water‐operational integrated circuits for long‐term biosensing systems and energy‐efficient brain‐inspired computing.

## Graphical abstract

Four NDI‐2Tz copolymers (P‐XO, X = 3–6) with modified EG side chains are synthesized. They exhibit exceptional n‐type operational stability (over 60 h) in OECTs, fast switching (<10 ms), and excellent ambipolar behavior. Increasing the length of side chains enhances the hydrophilicity of the materials, thereby boosting electrochemical doping efficiency. This study reveals EG substitution's impact on NDI‐2Tz copolymers, providing insight for stable, low‐power n‐type/ambipolar materials.

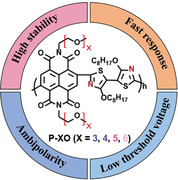

## 1 Introduction

Organic electrochemical transistors (OECTs) have ascended as rising stars, demonstrating tremendous potential in neuromorphic computing,^[^ ^1^, ^2^, ^3^, ^4^, ^5^ ^]^ bioelectronics,^[^ ^6^, ^7^, ^8^ ^]^ and stretchable/wearable electronics.^[^ ^9^, ^10^, ^11^ ^]^ The OECTs efficiently transduce ionic signals into electrical ones. The ion injection into the active channel under gate bias facilitates the electrochemical doping/dedoping of the active channel by charge compensation.^[^ ^12^ ^]^ The channel materials of the OECT are formed by organic mixed ionic‐electronic conductors (OMIECs), which possess the dual ability to transport ions and electrons. Within the OECT architecture, these materials function either in the aqueous solution or ionic liquid.^[^ ^13^ ^]^ Due to the coupling of ionic and electronic transport, OMIECs exhibit a high volumetric capacitance, resulting in higher transconductance of transistors (>1 mS) and lower operating voltage (<1 V) in comparison to field‐effect transistors.^[^ ^14^ ^]^ The unique benefits endow OECTs with the ability to mimic biological events, such as neuromorphic computing and biomarker sensing at the biological interface.^[^ ^7^, ^15^, ^16^ ^]^

The ion‐to‐electron transduction between OMIECs and electrolytes is associated with the OECT transconductance *g*_m_, a crucial parameter for signal amplification, indicating how sensitive the output current is to a change of the input voltage,^[^ ^17^ ^]^ which is calculated based on the following equation.^[^ ^18^ ^]^

(1) $$g_{m}=\partial I_{D}\partial V_{G}=\frac{\mathrm{Wd}}{L}\mu C^{*}\left(V_{G}-V_{\mathrm{th}}\right)$$

where *I_D_* is the drain current, *V_G_* is the gate voltage, *V_th_* is the threshold voltage, and *W*, *d*, and *L* are the channel width, thickness, and length, respectively. Moreover, *g*_m_ is proportional to the intrinsic OMIECs property µC^*^, where *µ* is the charge carrier mobility and C^*^ is the volumetric capacitance. Furthermore, other characteristics of OECTs, including threshold voltage, operation speed, and stability, need to be considered for performance improvement.^[^ ^19^ ^]^ The most commonly used OMIEC for OECTs is the p‐type poly(3,4‐ethylenedioxythiophene)‐poly(styrenesulfonate) (PEDOT: PSS), which can achieve a *g_m_* in the order of mS and has been successfully applied in numerous bioelectronic applications.^[^ ^20^ ^]^

It was reported that the introduction of the hydrophilic and polar EG side chains to the polymer backbone to promote ion transport in OMIECs for both p‐type (hole‐transporting, doped by anions) and n‐type (electron‐transporting, doped by cations).^[^ ^21^, ^22^ ^]^ One crucial p‐type semiconducting (accumulation mode) OMIEC example is bithiophene‐thienothiophene copolymer bearing EG side chains (Pg2T‐TT), which is widely used to demonstrate OECT applications in stretchable electronics and neuromorphic devices.^[^ ^9^ ^]^ However, the development of n‐type OMIECs falls much behind the p‐type ones. Poly(benzimidazobenzophenanthroline) (BBL) is an excellent fused, ladder‐type, and electron‐deficient n‐type OMIEC, with a highly planar, rigid backbone and delocalized charge carriers.^[^ ^23^, ^24^ ^]^ Most n‐type polymers are weak donor‐acceptor (D‐A) copolymers, such as NDI‐thiophene‐based P90,^[^ ^25^ ^]^ diketopyrrolopyrrole‐thiophene‐based 2DPP‐OD‐TEG ^[^ ^26^ ^]^ and DPP‐g2T,^[^ ^27^ ^]^ or bithiophene diimide‐based f‐BTI2TEG‐FT.^[^ ^28^ ^]^ Another polymer, PgNaN, is of acceptor–acceptor (A–A) type.^[^ ^29^ ^]^ Small molecule n‐type OMIECs are worth investigating due to their high purity and negligible batch‐to‐batch variations. Ginger et al. reported a fullerene‐based C60‐TEG,^[^ ^30^ ^]^ and Wan et al. have developed a series of naphthalene bis‐isatin and rhodamine‐based gNR,^[^ ^31^ ^]^ hgNR,^[^ ^31^ ^]^ and 3gDNR.^[^ ^32^ ^]^

Recently, many polymers with high µC* values (such as f‐BseI2g‐SVSCN,^[^ ^33^ ^]^ p(C2F‐V,^[^ ^34^ ^]^ PANDA2 ^[^ ^35^ ^]^) have been reported. However, the existing research rarely focuses on the operational stability of devices. Applications such as logic circuits, electrophysiological signal recording, and neuromorphic computing require devices to have long‐term operational stability. Unfortunately, most current materials lack this characteristic, thus limiting the practical application of OECTs. The stability of n‐type materials is primarily influenced by chemical, photochemical, and electrochemical factors.^[^ ^36^ ^]^ Air sensitivity is also an important factor.^[^ ^37^ ^]^ The thermodynamic stability of oxidation‐reduction reactions involving these environmental substances is closely related to their lowest unoccupied molecular orbital (LUMO) energy levels. The LUMO of n‐type OMIECs must be lower than −4 eV to avoid electrochemical side reactions like the oxygen reduction reaction (ORR).^[^ ^36^ ^]^ Previously, our team reported a type of non‐fused planar naphthalenediimide (NDI)‐dialkoxybithiazole (2Tz)‐based copolymers (P‐3O) for OECTs.^[^ ^38^ ^]^ which exhibits excellent performance with ambipolarity and remarkable stability. However, P‐3O‐based OECTs’ threshold voltage is approximately three times that of BBL,^[^ ^23^ ^]^ which limits the potential application of P‐3O. The backbone's deep LUMO level and high planarity and rigidity make NDI‐2Tz copolymers a good platform for further OECT applications. It is essential to tailor ion‐electron correlations to improve the transport properties of n‐type NDI‐2Tz copolymers. Recently, research works ^[^ ^39^, ^40^, ^41^, ^42^ ^]^ have highlighted that increasing the volume of polar EG side chains would make passive swelling and bulk doping easier, which might reduce the barrier to the threshold voltage for OECTs.

Inspired by these results, we customized non‐fused NDI‐2Tz copolymers by systematically increasing the length of polar EG side chains and studying their performance in OECTs. All NDI‐2Tz conjugated copolymers‐based OECTs display remarkable ambipolar characteristics, which allows for both p‐ and n‐type operations. Most excitingly, all OECT devices operate for over 60 h in water without significant degradation and maintain their high on‐off ratio. From P‐3O to P‐6O, we found that by increasing the length of polar EG side chains, the threshold voltage was remarkably reduced to 0.27 V due to the increased hydrophilic nature of these polymers. This work sheds light on the design of exceptional n‐type/ambipolar materials for OECTs. It demonstrates the potential of incorporating these ambipolar polymers into water‐operational integrated circuits for long‐term biosensing systems and energy‐efficient brain‐inspired computing.

## 2 Results

### 2.1 Aqueous Electrolyte Gated OECTs

**Figure 1:**
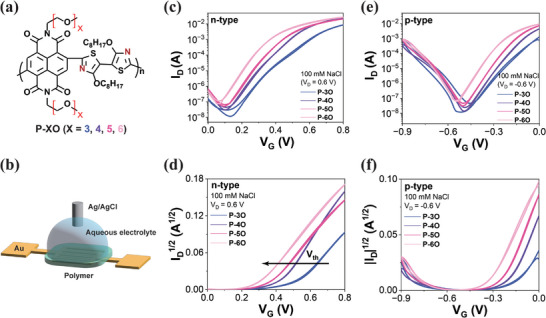
a) Chemical structures of the NDI‐2Tz copolymers. b) Schematic of OECT device (Channel width/length = 10.62 cm/5 µm). c) n‐type transfer curve (*V*_*D*_ = 0.6 V), d) *I*_*D*_^1/2^‐*V*_*G*_ plot of n‐type, e) p‐type transfer curve (*V*_*D*_ = −0.6 V), f) *I*_*D*_^1/2^‐*V*_*G*_ plot of p‐type of OECT devices with 100 mm NaCl aqueous electrolytes.

NDI‐2Tz copolymers with different EG chain lengths (P‐3O, P‐4O, P‐5O, and P‐6O, shown in **Figure** **1**a) were employed as the channel materials to fabricate OECTs by employing interdigitated electrodes as source and drain. The synthesis process of monomers and their corresponding polymers and the characterization of their relevant properties are detailed in Schemes S1 and S2 and Figures S1–S12 (Supporting Information). The device architecture is illustrated in Figure 1b. To assess the electrochemical properties of the polymers, a 100 mM NaCl aqueous solution was applied as the electrolyte, while an Ag/AgCl electrode served as the gate. The conjugated polymers were dissolved in chloroform and subsequently deposited via spin coating. Subsequently, the deposited films were annealed on the hot plate to enhance adhesion between the polymer film and substrate.

The OECT performance of P‐3O, P‐4O, P‐5O, and P‐6O were investigated, and the corresponding transfer and output curves are given in Figure 1 and Figure S13 (Supporting Information). Notably, all these polymers exhibit ambipolar transistor features operating in accumulation mode. The transistors exhibit an exceptional on‐off ratio of 10^5^ in n‐type mode (Figure 1c), which is outstanding compared with reported n‐type polymers.^[^ ^33^, ^43^, ^44^, ^45^ ^]^ An observed trend indicates that the threshold voltage shifts toward smaller values with an increase in the chain length of EG, as shown in Figure 1d and **Table** **1**. This trend agrees with the hydrophilic nature of these polymers, which is evident in the decrease of water contact angles from P‐3O to P‐6O (**Table** **2**; Figure S14, Supporting Information). Additionally, with an increase in side‐chain length, changes in the morphology of the polymer films are evident. The AFM test results in Figure S15 (Supporting Information) reveal that these polymer films exhibit a fibrous structure. The p‐type transistors exhibit an on‐off ratio of ≈10^4^ (Figure 1e), and the threshold voltage is almost unchanged with the length of the EG side chains (Figure 1f). The EG chains are attached to the electron‐deficient NDI moiety. In contrast, the hydrophobic alkyl chains substitute on the dialkoxybithiazole thiazole moiety. This distinction may be attributed to the hindrance of anionic doping and the suppression of p‐type behavior by alkyl side chains. In addition, the output curves of these polymers exhibit no significant hysteresis during forward and reverse scans. In the p‐type output curves, a diode‐like characteristic is observed at low gate voltages, where the *I*_*D*_ increases exponentially with the *V*_*D*_. This result is also observed in 2DPP‐OD‐TEG, where the channel region near the drain is doped with n‐type carriers (electron accumulation) when *V*_*D*_ < *V*_*G*_ – *V*_*th,n*_.^[^ ^26^ ^]^ By plotting *g*_*m*_ against (*V*_*G*_‐*V*_*th*_)*Wd*/*L* curves, as shown in Figure S16 (Supporting Information), we extracted the maximum µC* values of 1.309 F V^−1^ cm^−1^ s^−1^ for P‐3O, 0.925 F V^−1^ cm^−1^ s^−1^ for P‐4O, 0.916 F V^−1^ cm^−1^ s^−1^ for P‐5O, and 0.634 F V^−1^ cm^−1^ s^−1^ for P‐6O. These µC* values are comparable to BBL.^[^ ^23^ ^]^

**Table 1:** Aqueous electrolyte‐gated n‐type OECTs characteristics based on the EG substituted NDI‐2Tz copolymers (three devices were measured for each polymer to extract the standard deviation).

Polymer	Thickness [nm]	On/Off ratio	*V*_*th*_ [V]	*g*_*m*_ [S]	*g*_*m,norm*_ [S cm^−1^]	*τ*_*on*_ [ms]	*τ*_*off*_ [ms]
P‐3O	58	>10^4^	0.49 ± 0.02	0.05 ± 0.01	0.41 ± 0.07	8.2 ± 0.29	1.1 ± 0.14
P‐4O	54	>10^5^	0.40 ± 0.02	0.089 ± 0.03	0.77 ± 0.29	2.2 ± 0.91	1.2 ± 0.38
P‐5O	63	>10^5^	0.34 ± 0.02	0.068 ± 0.01	0.51 ± 0.10	6.2 ± 2.90	0.9 ± 0.06
P‐6O	64	>10^5^	0.27 ± 0.01	0.084 ± 0.01	0.61 ± 0.17	2.8 ± 0.75	1.2 ± 0.44

**Table 2:** Optical properties, electrochemical properties, energy levels, and water contact angle (θ) of the EG substituted NDI‐2Tz polymers.

Polymer	λ_onset_, ^film^ [nm]	E_g_^opt.^ ^a)^ [eV]	HOMO^b)^ [eV]	LUMO^c)^ [eV]	E_onset_^ox.^ ^b)^ [V]	E_onset_^red.^ ^c)^ [V]	Θ [°]
P‐3O	1225	1.01	−5.58	−4.30	0.48	−0.80	83.4 ± 0.7
P‐4O	1259	0.99	−5.57	−4.37	0.47	−0.73	74.8 ± 0.6
P‐5O	1294	0.96	−5.59	−4.36	0.49	−0.74	64.7 ± 1.0
P‐6O	1308	0.95	−5.54	−4.37	0.44	−0.73	63.9 ± 1.3

^a)^ Optical bandgap from the onset of the thin film absorption spectra;

^b)^ Onset of oxidation from CV recorded in a CHCN_3_ solution containing Bu_4_NPF_6_ electrolyte (0.1 mol L^−1^). HOMO = −(5.10 + *E*_onset_^ox.^) eV;

^c)^ Onset of reduction from CV recorded in a CHCN_3_ solution containing Bu_4_NPF_6_ electrolyte (0.1 mol L^−1^). LUMO = −(5.10 + *E*_onset_^red.^) eV (Figure S24, Supporting Information).

**Figure 2:**
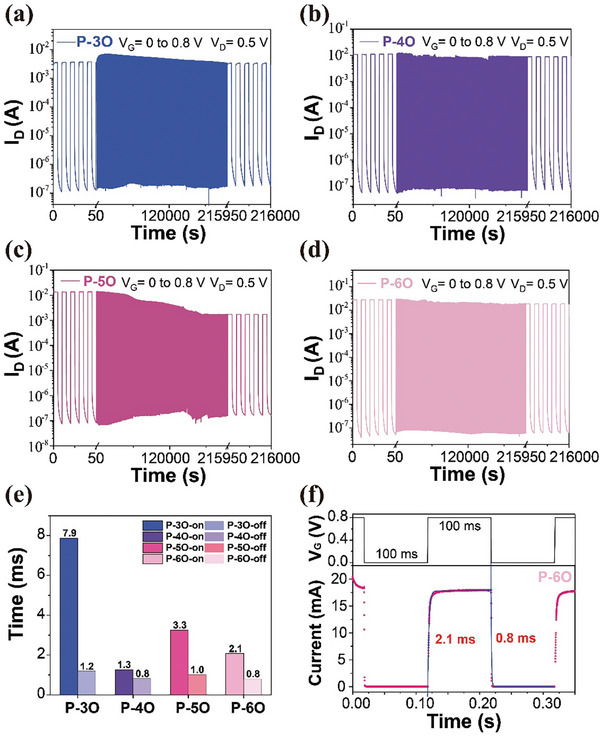
Comparison of OECTs n‐type operation stability a) P‐3O b) P‐4O c) P‐5O d) P‐6O. The drain current (*I_D_*) was monitored over 21600 pulse cycles, during which a gate voltage pulse *V_G_* = 0.8 V was applied for 5 s with an interval time of 5 s (*V_D_* = 0.5 V). e) N‐type transient response time *τ*_*on/off*_. f) The current response to the gate voltage of P‐6O in the n‐type mode in 100 mm NaCl aqueous solution.

These polymers exhibit remarkable stability when operating in an n‐type mode in water, retaining their function over 1000 cycles without any performance degradation (Figure S17, Supporting Information). It is noteworthy that, except for P‐5O, which experiences a reduction in the on‐off ratio by order of magnitude after operating for an extended period (Figure 2c). all other polymers consistently maintain the same order of magnitude for the on‐off ratio for a duration exceeding 60 h (Figure **2**a–d). Mainly, P‐6O‐based OECTs maintain an on‐off ratio more significant than 10^5^ (Figure 2d), which has not been reported for n‐type organic materials. We utilized the same data set of P‐3O (Figure 2a) from previous work.^[^ ^38^ ^]^ To confirm that the remarkable stability of these devices is not attributed to the interdigitated electrode structure, we conducted validation using electrodes with a single channel size of 3000/200 µm (*W*/*L*). The P‐4O‐based OECT exhibited excellent stability during continuous 60 h testing as well (shown in Figure S18, Supporting Information). Furthermore, all the devices respond rapidly to the gate voltage, which switches on and off with a transient response time (*τ*_*on/off*_) of less than 10 ms (Figure 2e); these transient response values are comparable to BBL and NDI‐T2 polymers.^[^ ^23^, ^46^ ^]^ P‐3O shows the slowest switch‐on time and requires more than 200 on‐ and off‐cycles to reach the maximum output current, indicating that the hydration of the P‐3O polymer film occurs more slowly than that of the other polymers. The characteristics of the P‐6O device‘s transient response are given in Figure 2f. An exponential function fits the *I_D_* curve; the obtained time constants are *τ*_*on* */off*_ = 2.1/0.8 ms.

**Figure 3:**
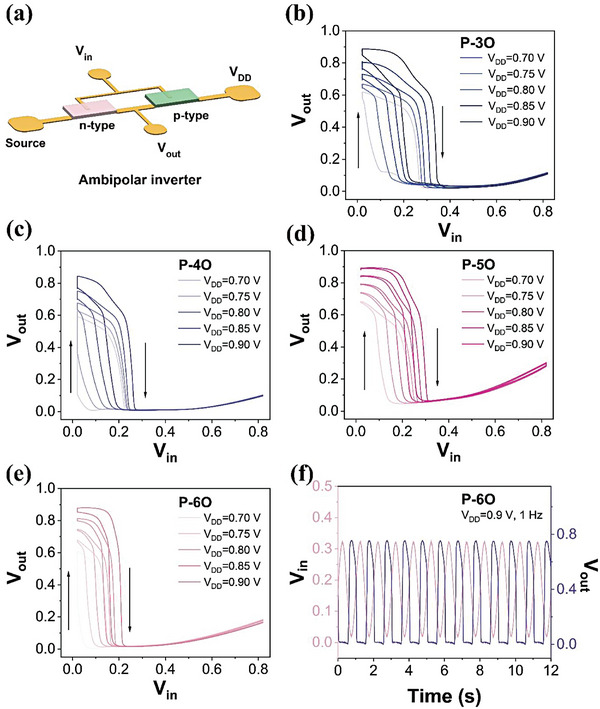
a) Schematic of an ambipolar inverter. Inverter characteristics of b) P‐3O, c) P‐4O, d) P‐5O, e) P‐6O. f) The input–output characteristics of an inverter where the input signal is a sinewave between 0 and 0.3 V with a frequency of 1 Hz.

Furthermore, ambipolar inverters employing a single polymer material are fabricated and operated in a 100 mm NaCl electrolyte (**Figure** **3**a). Developing complementary inverters is essential for logic circuits or biosignal amplification, as it necessitates the integration of both p‐ and n‐type transistors. Ambipolar polymers offer the advantage of using a single material for constructing an integrated circuit, significantly reducing fabrication complexity. The characteristics of ambipolar inverters based on P‐3O, P‐4O, P‐5O, and P‐6O are shown in Figure S19 (Supporting Information) and Figure 3b–e, respectively. Both forward and backward scans were evaluated, and the hysteresis of the inverters decreased with increasing EG sidechains. Consequently, P‐6O shows the smallest hysteresis. At the same time, the P‐6O‐based inverter switches ≈0.2 V, representing the lowest switching voltage among all polymers. This trend aligns with the threshold voltage of n‐type OECTs and the observed hysteresis, indicating that longer EG side chains improve ion‐electron coupling efficiency and accelerate device response speed. Additionally, all inverters achieved peak voltage gain at *V*_*DD*_ = 0.9 V, with *V*_*in*_ values of 0.34 V for P‐3O, 0.25 V for P‐4O, 0.29 V for P‐5O, and 0.21 V for P‐6O. The maximum gains are 22.5, 17.5, 18.5, and 27.4 V/V, respectively (Figure S20, Supporting Information). These gain values are comparable to the reported gains of inverters based on single‐component ambipolar materials.^[^ ^26^, ^38^, ^47^ ^]^ Figure 3f demonstrates that the P‐6O inverter oscillates with *V*_*in*_ between 0 and 0.3 V at 1 Hz.

### 2.2 Characterization of the Polymers

The impact of the EG side chains on the reduction and oxidation potential in aqueous conditions was studied by measuring the cyclic voltammetry (CV) of the polymers on indium tin oxide (ITO) substrates in a 100 mm NaCl aqueous solution; it was observed that the reduction onset potentials decrease with increasing EG length while the oxidation onset potentials are almost identical for all four polymers. The reduction onset potentials are −0.32 V for P‐3O, −0.32 V for P‐4O, −0.29 V for P‐5O, and −0.27 V for P‐6O (Figure S21a, Supporting Information). According to previous studies, this indicates that a more hydrophilic environment can shift the reduction potential of conjugated polymers to smaller values.^[^ ^48^ ^]^ Furthermore, the oxidation onset potentials are recorded as 0.69 V for P‐3O, 0.70 V for P‐4O, 0.69 V for P‐5O, and 0.66 V for P‐6O, as shown in Figure S21b (Supporting Information). The reduced reduction potential can be ascribed to the hydrophilic EG side chains on the electron‐withdrawing moiety NDI, which facilitates the penetration of ions into the polymer film and doping. Therefore, the shift in reduction onset indicates increasing EG length assists electrochemical doping at a lower potential. On the other hand, anionic doping is dependent on the hydrophobic alkyl side chains located on the donor dialkoxybithiazole moiety, which remain constant, leading to no significant change in the oxidation potential. The effect of the EG side chain on these polymers’ hydrophilicity is investigated by water contact angle (Figure S14, Supporting Information). The contact angle decreases as the length of EG side chains increases, indicating that P‐6O has the best wettability that enables efficient ion diffusion and electrochemical doping, consistent with the aqueous CV data.

The electrochemical spectroscopy demonstrates the absorbance spectra of P‐3O, P‐4O, P‐5O, and P‐6O films in the reduced states at various bias potentials ranging from 0 to 0.8 V (Figure S22, Supporting Information). The intramolecular charge transfer (ICT) absorption between the strong acceptor NDI and the weak donor dialkoxybithiazole (≈950 nm) gradually diminishes with the reducing potential. Moreover, when a negative potential is applied, new absorption features can be observed between 550 and 650 nm, and the intensity of these features gradually increases with higher negative voltages. The emergence of new polaronic absorption features occurs between 1100 and 1200 nm. However, due to the limited detection range of the detector, performance characteristics above 1200 nm cannot be explored. These results confirm that cations effectively dope the polymer films upon the reducing potential, which aligns with our previous findings on NDI‐2Tz copolymers.^[^ ^42^ ^]^

To evaluate the effect of the length of EG side chains on the electronic properties of these polymers, we measured the absorption on pristine polymer films and CV in acetonitrile. The bandgap, HOMO and LUMO were calculated and summarized in Table 2. In addition, density functional theory (DFT) calculations on the monomer units are included in Figure S23 (Supporting Information), which shows that the energy level differences among these polymers are insignificant. Based on these results, we can exclude the influence of EG side chain length on the energy level.

**Figure 4:**
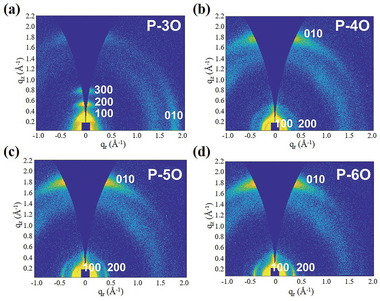
2D GIWAXS patterns of a) P‐3O, b) P‐4O, c) P‐5O, and d) P‐6O thin films.

The polymer thin film morphologies were investigated using grazing incidence wide‐angle X‐ray scattering (GIWAXS) to understand how the EG chain length influences molecular packing. The results are displayed in **Figures** **4**a–d and S25 (Supporting Information), which includes the corresponding horizontal and vertical line cuts. The EG chain length plays a vital role in altering the microstructure and molecular packing despite all these polymers having the same NDI‐2Tz backbone. Specifically, P‐3O adopts a predominant edge‐on orientation with lamellar diffractions up to the third order (300) at *q*_*z*_ = 0.78 Å^−1^ in the out‐of‐plane (OOP) direction and a π–π stacking (010) peak at *q*_*r*_ = 1.81 Å^−1^ in the in‐plane (IP) direction. On the other hand, P‐4O, P‐5O, and P‐6O become dominated by face‐on bimodal packing, exhibiting lamellar diffractions up to the second order (200) at *q*_*r*_ = 0.44 Å^−1^, *q*_*r*_ = 0.43 Å^−1^, *q*_*r*_ = 0.40 Å^−1^, and a (010) peak at *q*_*z*_ = 1.82 Å^−1^, *q*_*z*_ = 1.84 Å^−1^, *q*_*z*_ = 1.82 Å^−1^. By drawing insights from references,^[^ ^49^, ^50^, ^51^, ^52^ ^]^ we can deduce that the side chain length elongation enhances self‐interaction, thus altering the dominance of polymer backbone interactions (π–π stacking). The interplay of molecular interactions governs the face‐on versus edge‐on arrangement. Consequently, a shift from an edge‐on to a face‐on orientation was noted with the rise in EG side chain length. The π–π stacking at d‐spacing (*d* = 2π/*q*) was calculated to be 3.47 Å for P‐3O, 3.45 Å for P‐4O, and 3.41 Å for P‐5O, and 3.45 Å for P‐6O from the (010) diffraction, respectively.

Meanwhile, to reveal differences in their crystallinity, we calculated their coherence lengths using the Scherrer equation (CL = 2π*K*/ZFWHW, *K *= 0.89) and paracrystalline disorder parameters (*g* = SQRT(FWHM/2π*q*) based on the full width at half maximum (FWHM) and center position (*q*) of the 010 diffraction peak for the four polymers. As shown in Table S1 (Supporting Information), with the increase in side chain length, the *π*–*π* stacking of polymers becomes denser, which might facilitate higher electron mobility.^[^ ^32^ ^]^ However, longer side chains also lead to a decrease in the ordering of polymer molecular arrangements. While the edge‐on orientation, high crystalline coherence length (CCL), and low paracrystalline disorder in P‐3O are beneficial for lateral charge carrier transport in OECTs,^[^ ^32^, ^53^ ^]^ the performance of OECTs is influenced by multiple factors. The molecular packing orientation does not solely determine it. Other factors, such as ion injection, mobility, and ion‐electron coupling efficiency, also play significant roles. The superior performance of P‐6O in OECT results from a balanced combination of charge carrier mobility, ion mobility, and ion‐electron coupling efficiency.

## 3 Conclusion

We have synthesized a series of NDI‐2Tz copolymers substituted with EG side chains of increasing length and studied their performance in aqueous electrolyte‐gated electrochemical transistors. All these copolymers exhibit high stability in n‐type mode, fast operation speed (<10 ms), low threshold voltage, and ambipolarity. The NDI‐2Tz backbone substituted hexa(ethylene glycol) (P‐6O) performs the best in aqueous electrolyte gated OECTs, which exhibit the lowest threshold voltage (0.27 V), ultra‐high stability (operating in water over 60 h while maintaining 10^5^ on‐off ratio). Further, we fabricated the aqueous electrolyte‐gated ambipolar inverters utilizing the ambipolar copolymers. The P‐6O‐based inverter can transduce a 0.2 V input signal to 0 V output, the lowest input voltage among all the polymers, corresponding to the trend observed in the threshold voltages. This work reveals the structure‐property relationship of NDI‐2Tz copolymers substituted with EG side chains, providing insight for designing ambipolar materials for highly stable and low‐power organic electrochemical transistors.

## 4 Experimental Section

### Materials

The synthesis details of the NDI‐2Tz‐based D‐A copolymers are described in the Supporting Information. Chloroform, 1‐ethyl‐3‐methylimidazolium bis(trifluorosulfonyl)imide, and poly(vinylidene fluoride‐co‐hexafluoropropylene) were purchased from Sigma–Aldrich and used as received. The interdigitated microelectrodes IDA‐Au‐6 were purchased from MicruX technologies (Number of feet: 30 pairs, Electrode width: 5 µm, Individual channel width: 1.8 mm).

### Device Fabrication and Characterizations

The polymer solutions were prepared in chloroform (5 mg mL^−1^). The interdigitated microelectrodes were treated with UV ozone for more than 15 min, following spin‐coating the polymer solutions at 1000 rpm for 30 s. Subsequently, the deposited films were annealed on the hot plate at 100 °C for 15 min. The electrical characterization of OECTs was performed by a Keithley source‐meter 2602B. The inverters were fabricated by connecting two OECTs via probe stations and measured by a Source Measure Unit (SMU) customized by Cici Research. The response time testing of OECTs was conducted by applying a pulsed voltage with a frequency of 5 Hz and an amplitude of 0.8 V to the gate electrode using an arbitrary function generator. In contrast, a constant voltage of 0.5 V was applied between the source and drain using a Keithley 2612B to measure the current variation.

### Cyclic Voltammetry (CV)

Cyclic voltammetry measurements were performed using a CHI760E Electrochemical Workstation (Shanghai Chenhua Instruments Co. Ltd.) with a standard three‐electrode configuration in 100 mm NaCl aqueous solution. Polymer thin films were spun on ITO glass employed as a working electrode and used together with a platinum counter electrode and an Ag/AgCl reference electrode.

### UV–Vis–NIR Electrochemical Spectroscopy

The polymer thin films were deposited on ITO glass and then immersed into a cuvette filled with 100 mm NaCl aqueous electrolyte, together with an Ag/AgCl electrode. The UV–vis–NIR transmission of polymer films was monitored in situ at different voltages (vs Ag/AgCl) to probe the neutral and charged states during electrochemical doping.

### Film‐Thickness Measurements

The thickness of spun polymer thin films was characterized by a Veeco Dektak 150 profilometer.

### GIWAXS

GIWAXS experiments were conducted on a GANESHA 300XL+ system from JJ X‐ray. The instrument was equipped with a Pilatus 300K detector, with a pixel size of 172  × 172 µm. The X‐ray source is a Genix 3D Microfocus Sealed Tube X‐Ray Cu‐source with an integrated Monochromator (multilayer optic “3D version” optimized for SAXS) (30 W). The wavelength used was *λ* = 1.5408 Å. The detector moved in a vacuum chamber with a sample‐to‐detector distance between 0.115 and 1.47 m, depending on the configuration used, as calibrated using silver behenate (d001 = 58.38 Å). The minimized background scattering plus high‐performance detector allowed for a detectable q‐range varying from 3 × 10^−3^ to 3 Å^−1^ (0.2 to 210 nm). The sample was placed vertically on the goniometer and tilted to a glancing angle of 0.2° with respect to the incoming beam. The accumulation time was 2 h for each measurement.

### Atomic Force Microscopy

The AFM measurements were conducted in tapping mode using a Bruker Nano Inc with a SCANSYST‐AIR probe. The scanning area was set to 20 µm, and the measurements were performed at ≈25 °C room temperature. Data analysis was done utilizing NanoScope Analysis 3.00 (Bruker Nano Analytics).

## Conflict of Interest

The authors declare no conflict of interest.

## Supporting information

Supporting Information

## Data Availability

The data that support the findings of this study are available from the corresponding author upon reasonable request.
