# Supplementary material for: Over 60 h of Stable Water‐Operation for N‐Type Organic Electrochemical Transistors with Fast Response and Ambipolarity
Source: Adv Sci (Weinh). 2024 May 29;11(29):2400872. doi: 10.1002/advs.202400872 (PMC11304290; doi:10.1002/advs.202400872)
Supplement: Supplementary file 1 — Supporting Information [file ADVS-11-2400872-s001.docx]

Supporting Information

**Over 60 Hours of Stable Water-Operation for N-Type Organic Electrochemical Transistors with Fast Response and Ambipolarity**

Tao Pan^†^, Xinnian Jiang^†^, Eveline R. W. van Doremaele^†^, Junyu Li, Tom P. A. van der Pol, Chenshuai Yan, Gang Ye*, Jian Liu, Wenjing Hong, Ryan C. Chiechi, Yoeri van de Burgt*, Yanxi Zhang*

Tao Pan (equal contribution), Xinnian Jiang (equal contribution), Chenshuai Yan, Wenjing Hong, Yanxi Zhang

The Institute of Flexible Electronics (IFE, Future Technologies) & IKKEM & State Key Laboratory of Physical Chemistry of Solid Surfaces, College of Chemistry and Chemical Engineering, Xiamen University, Xiamen 361005, China.

E-mail: [ifeyxzhang@xmu.edu.cn](mailto:ifeyxzhang@xmu.edu.cn)

Eveline R. W. van Doremaele (equal contribution), Yoeri van d Burgt

Microsystems, Department of Mechanical Engineering & Institute for Complex Molecular Systems, Eindhoven University of Technology, 5600 MB, Eindhoven, The Netherlands.

E-mail: [y.b.v.d.burgt@tue.nl](mailto:y.b.v.d.burgt@tue.nl)

Junyu Li

Sinopec Shanghai Research Institute of Petrochemical Technology, Shanghai 201028.

Tom P. A. van der Pol

Molecular Materials and Nanosystems & Institute for Complex Molecular Systems, Eindhoven University of Technology, Eindhoven 5600 MB, The Netherlands.

Gang Ye

Key Laboratory for the Green Preparation and Application of Functional Materials, Hubei Key Laboratory of Polymer Materials, School of Materials Science and Engineering, Hubei University, Youyi Road 368, Wuhan, 430062 China

E-mail: [g.ye0612@ciac.ac.cn](mailto:g.ye0612@ciac.ac.cn)

Jian Liu

State Key Laboratory of Polymer Physics and Chemistry, Changchun Institute of Applied Chemistry, Chinese Academy of Science, Changchun, Jilin, 130022, P. R. China.

Ryan C. Chiechi

Department of Chemistry & Organic and Carbon Electronics Cluster, North Carolina State University, Raleigh, North Carolina 27695-8204, United States.

1. **Synthesis and Characterization of Materials**

**1.1 Reagents**

All reagents and solvents were commercial and were used as received. 2,6-dibromo-1,4,5,8-naphthalene tetracarboxylic diimide were purchased from TCI. 4,4'-bis(octyloxy)-2,2'-bis(trimethylstannyl)-5,5'-bithiazole (2Tz) were purchased from SunaTech. 2,5,8,11-tetraoxatridecan-13-amine and 2,5,8,11,14-pentaoxahexadecan-16-amine was synthesized according to the literature method.^[1]^ 4,9-dibromo-2,7-di(2,5,8,11-tetraoxatridecan-13-yl)benzo[lmn][3,8]phenanthroline-1,3,6,8(2H,7H)-tetraonewere (NDI-4O) and 4,9-dibromo-2,7-di(2,5,8,11,14-pentaoxahexadecan-16-yl) benzo[lmn][3,8]phenanthroline-1,3,6,8(2H,7H)-tetraone (NDI-5O) were synthesized according to literature procedures.^[2, 3]^ The Synthesis of P-3O^[4]^ and P-6O^[5]^ are reported in our recent work.

**1.2 Characterization**

^1^HNMR and ^13^CNMR were performed on a Varian Unity Plus (400 MHz) instrument at 25 ^◦^C, using tetramethylsilane (TMS) as an internal standard. NMR shifts are reported in ppm, relative to the residual protonated solvent signals of CDCl_3_ (δ= 7.26 ppm) or at the carbon absorption in CDCl_3_ (δ = 77.23 ppm). Multiplicities are denoted as singlet (s), doublet (d), triplet (t) and multiplet (m). High-Resolution Mass Spectroscopy (HRMS) was performed on a JEOL JMS 600 spectrometer. FT-IR spectra were recorded on a Nicolet Nexus FT-IR fitted with a Thermo Scientific Smart iTR sampler. GPC measurements were done on an Agilent GPC-PL220 room temperature GPC/SEC system at 30 °C *vs* polystyrene standards using hexafluoroisopropanol (HFIP) as eluent. Thermal properties of the polymers were determined on a TA Instruments DSC Q20 and a TGA Q50. DSC measurements were executed with two heating-cooling cycles with a scan rate of 10 °C min^−1^. TGA measurements were done from 20 to 800 °C with a heating rate of 20 °C min^−1^. Absorption spectra were recorded with a UV-vis-NIR spectrophotometer (Shimadzu UV3600). Cyclic voltammetry (CV) was carried out with a CHI760 Evoltammetric potentiostat in a three-electrode configuration where the working electrode was a platinum disk electrode, the counter electrode was a platinum wire, and the pseudo-reference was an Ag wire that was calibrated against ferrocene/ferrocenium redox (Fc/Fc^+^). Cyclic voltammograms for the D-A copolymer films deposited on the glassy carbon working electrode in CHCN_3_ solution containing Bu_4_NPF_6_ (0.1 mol L^-1^) electrolyte at a scanning rate of 50 mV s^-1^.


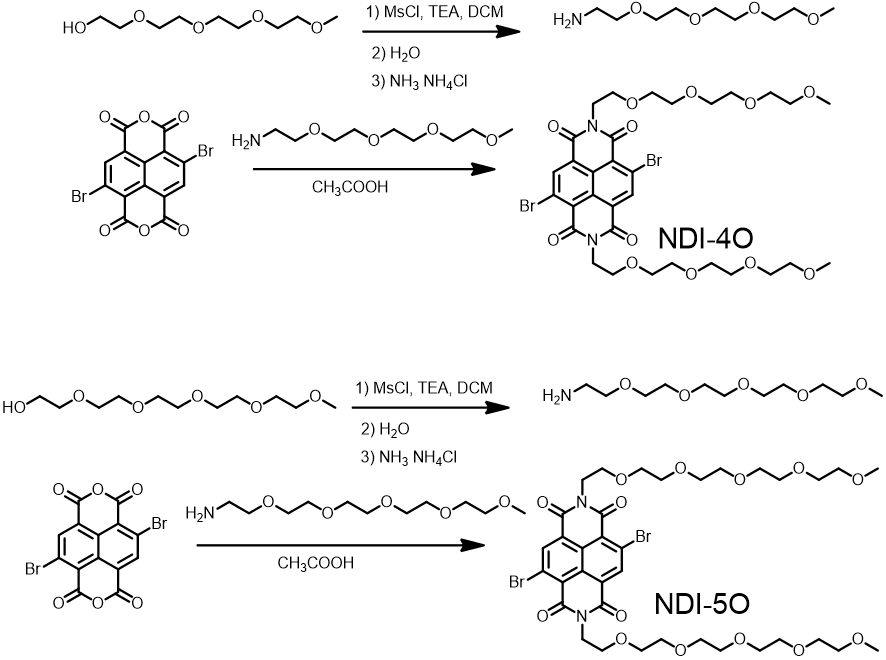


**Scheme S1.** Synthetic route for NDI-based monomers.

**1.3 Synthesis of NDI-4O**

2-4,9-dibromo-2,7-di(2,5,8,11-tetraoxatridecan-13-yl)benzo[lmn][3,8]phenanthroline-1,3,6,8(2H,7H)-tetraone 2,5,8,11-tetraoxatridecan-13-amine (770 mg, 3.4 mmol, 4 eq) was added to a suspension of 2,6-dibromo-1,4,5,8-naphthalene tetracarboxylic diimide (360 mg, 0.85 mmol, 1 eq) in 10 mL of glacial acetic acid and heated to 120°C for 4 h. The reaction mixture was then cooled to room temperature and added to water, then extracted with CH_2_Cl_2_. The organic phase was washed with brine, dried over anhydrous sodium sulfate and removed solvent by rotary evaporator. The crude product was purified by silica gel column chromatography with CH_2_Cl_2_/acetone as the eluent afforded the target compound (380 mg, 47%) as a bright yellow solid.

**NDI-4O**: ^1^HNMR (400 MHz, CDCl_3_): δ 8.97 (s, 2H), 4.46 (t, J = 5.6 Hz, 4H), 3.84 (t, J= 6.0 Hz, 4H), 3.73-3.67 (m, 4H), 3.64-3.56 (m, 16H), 3.54-3.43 (m, 4H), 3.35 (s, 6H). ^13^CNMR (100 MHz, CDCl_3_): δ 163.46, 163.37, 141.66, 130.97, 130.41, 127.97, 126.75, 74.54, 73.24, 73.19, 73.12, 72.71, 70.20, 61.65, 42.66. HRMS(ESI) Calcd. for C_32_H_40_Br_2_N_2_O_12_Na_1_ [M+Na]^+^: 827.08197, found: 827.08261.

**Figure S1.** ^1^HNMR spectra of NDI-4O.


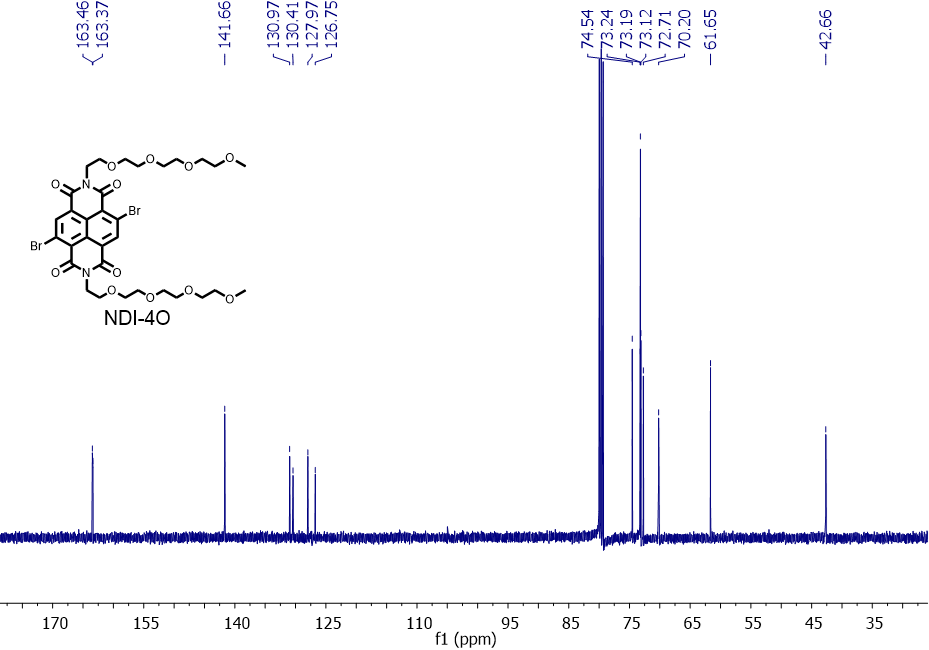


**Figure S2.** ^13^CNMR spectra of NDI-4O.

**Figure S3.** HRMS spectra of NDI-4O.

**1.4 Synthesis of NDI-5O**

4,9-dibromo-2,7-di(2,5,8,11,14-pentaoxahexadecan-16-yl)benzo[lmn][3,8]phenanthroline-1,3,6,8(2H,7H)-tetraone 2,5,8,11,14-pentaoxahexadecan-16-amine (230 mg, 0.92 mmol, 4 eq) was added to a suspension of 2,6-dibromo-1,4,5,8-naphthalene tetracarboxylic diimide (100 mg, 0.23 mmol, 1 eq) in 10 mL of glacial acetic acid and heated to 120 °C for 4 h. The reaction mixture was then cooled to room temperature and added to water (100 mL), then extracted with CH_2_Cl_2_. The organic phase was washed with brine, dried over anhydrous sodium sulfate and removed solvent by rotary evaporator. The crude product was purified by silica gel column chromatography with CH_2_Cl_2_/acetone as the eluent afforded the target compound (180 mg, 20%) as a bright yellow solid.

**NDI-5O**：^1^HNMR (400 MHz, CDCl_3_): δ 8.98 (s, 2H), 4.46 (t, J = 6 Hz, 4H), 3.85 (t, J = 6 Hz, 4H), 3.73-3.67 (m, 4H), 3.67-3.57 (m, 24H), 3.56-3.51 (m, 4H), 3.37 (s, 6H). ^13^CNMR(100 MHz, CDCl_3_): δ 163.49, 163.39, 141.68, 130.99, 130.43, 127.99, 126.77, 74.56, 73.23, 73.21, 73.20, 73.17, 73.13, 72.71, 70.22, 61.66, 42.67. HRMS(ESI) Calcd. for C_36_H_52_Br_2_N_3_O_14_ [M+NH_4_]^+^: 910.17901, found: 910.17683.

**Figure S4.** ^1^HNMR spectra of NDI-5O.

**Figure S5.** ^13^CNMR spectra of NDI-5O.


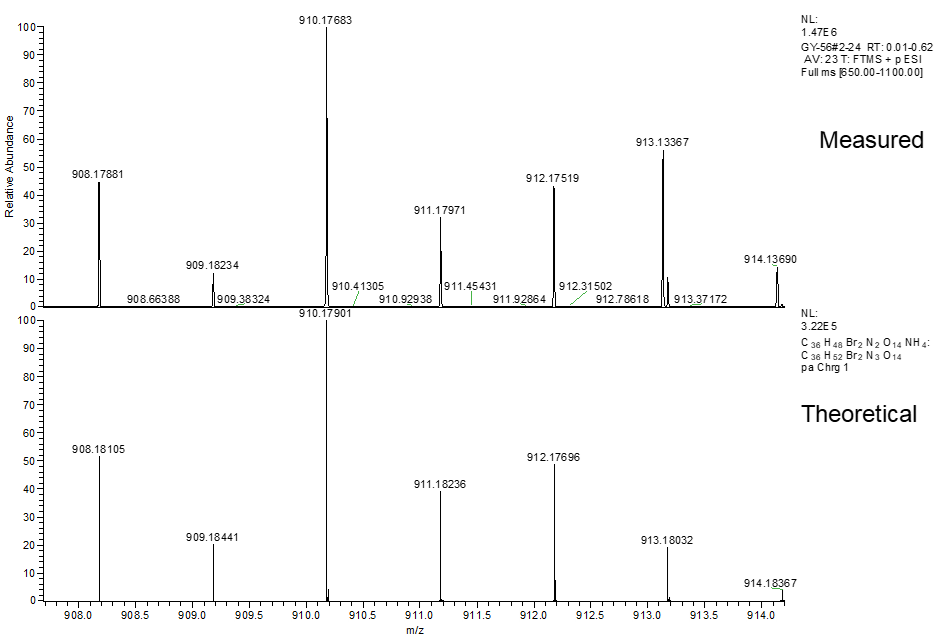


**Figure S6.** HRMS spectra of NDI-5O.


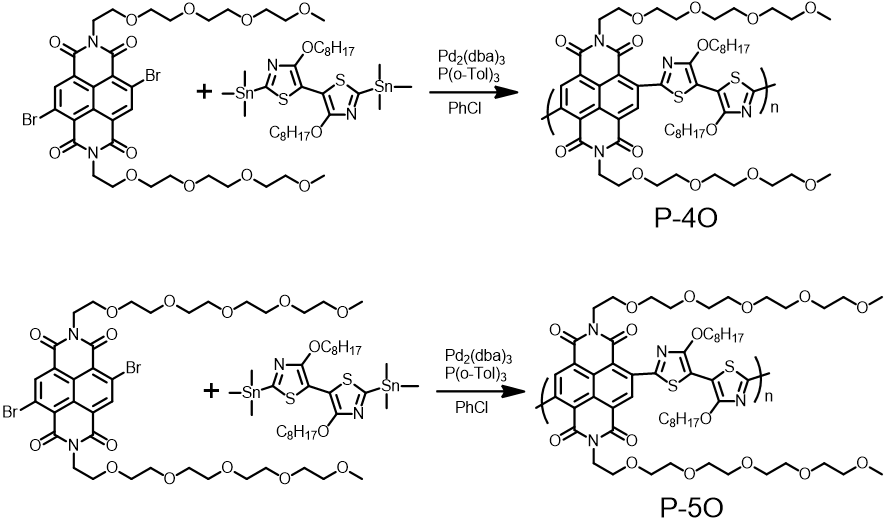


**Scheme S2.** Synthetic route for NDI-2Tz based low band gap conjugated polymers.

**1.5 General Synthetic Procedures for the NDI-2Tz-based Polymers**

To a dry three-neck flask, NDI-based monomer (0.1 mmol) and thiophene-based monomer (0.1 mmol) were added under argon followed by tris(dibenzylideneacetone) dipalladium [Pd_2_(dba)_3_] (8 mg) and tri(o-tolyl)phosphine [P(o-tolyl)_3_] (12 mg). The flask and its contents were subjected to 3 pump/purge cycles with N_2_ followed by the addition of anhydrous, degassed chlorobenzene (5 mL) via a syringe. The reaction mixture was stirred at 120 °C for three days. After cooling to room temperature, the deeply green-colored reaction mixture was dropped into 100 mL vigorously stirred methanol (containing 5 mL 12 M hydrochloride acid). After stirring for 4 hours, the precipitated solid was collected by filtration. The solid polymers were re-dissolved in chloroform and reprecipitated into methanol. After filtration, the polymers were subjected to sequential Soxhlet extraction. The sequential solvents were methanol, hexane and chloroform. Impurities and low-molecular-weight fractions were removed by methanol and hexane. Finally, the polymer solution in chloroform was concentrated to give the polymers as dark solid.

**P-4O:** Synthesis according to the general polymerization procedure: monomer NDI-4O (86.5 mg, 0.1 mmol), monomer 2Tz (75 mg, 0.1 mmol), dry chlorobenzene (5 mL). The polymer was obtained as a dark solid (102 mg, 96 %). ^1^HNMR (400 MHz, CDCl_3_): δ 10.72-8.01 (m, 2H), 5.26-3.01 (m, 42H), 2.31-0.71 (m, 30H). IR (cm^-1^): 692, 719, 764, 790, 896, 949, 982, 1065, 1103, 1176, 1209, 1245, 1297, 1314, 1366, 1441, 1487, 1564, 1646, 1698, 2854. GPC: Mn = 12.8 kDa, Mw = 15.8 kDa, PDI = 1.23.

**P-5O:** Synthesis according to the general polymerization procedure: monomer NDI-5O (71.4 mg, 0.08 mmol), monomer 2Tz (60 mg, 0.08 mmol), dry chlorobenzene (5 mL). The polymer was obtained as a dark solid (92 mg, 99 %). ^1^HNMR (400 MHz, CDCl_3_): δ 10.98-8.42 (m, 2H), 5.03-3.03 (m, 50H), 2.54-0.63 (m, 30H). IR (cm^-1^): 668, 692, 719, 763, 790, 896, 949, 982, 1065, 1103, 1176, 1208, 1245, 1297, 1314, 1366, 1441, 1487, 1564, 1647, 1698, 2854. GPC: Mn = 13.1 kDa, Mw = 17.1kDa, PDI = 1.30.


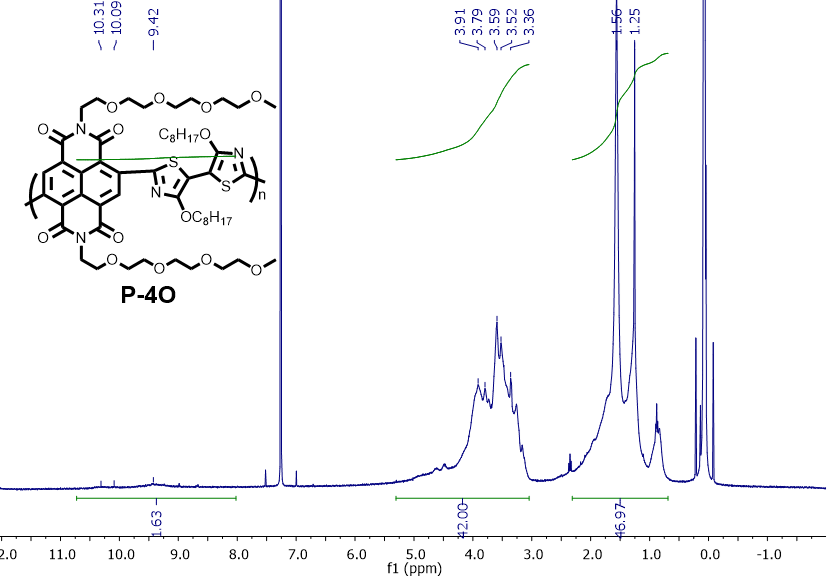


**Figure S7.** ^1^HNMR spectra of P-4O.


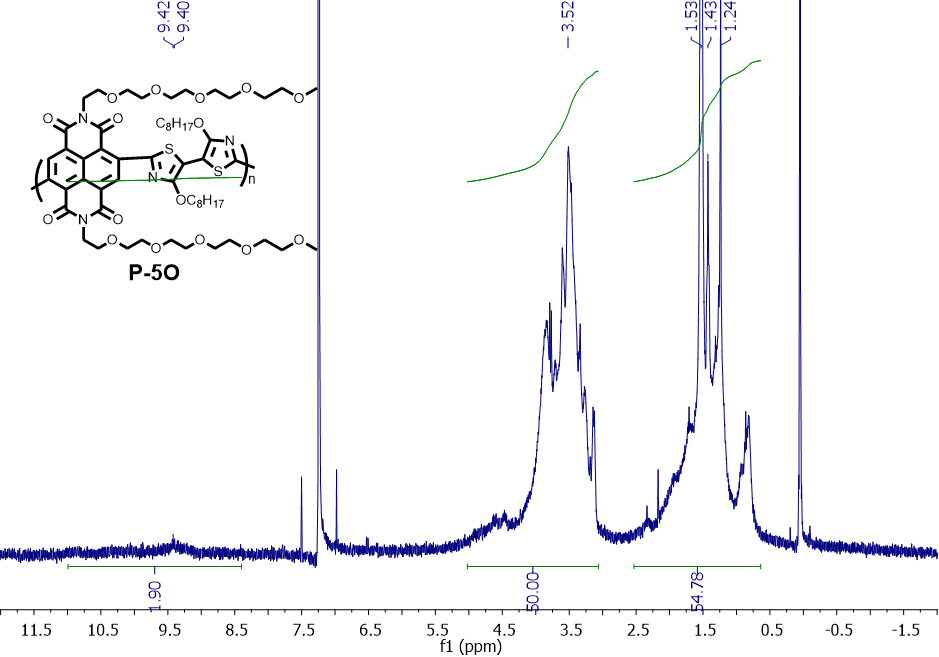


**Figure S8.** ^1^HNMR spectra of P-5O.


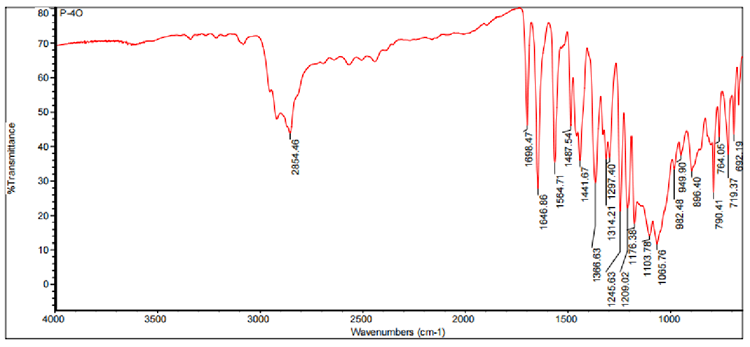


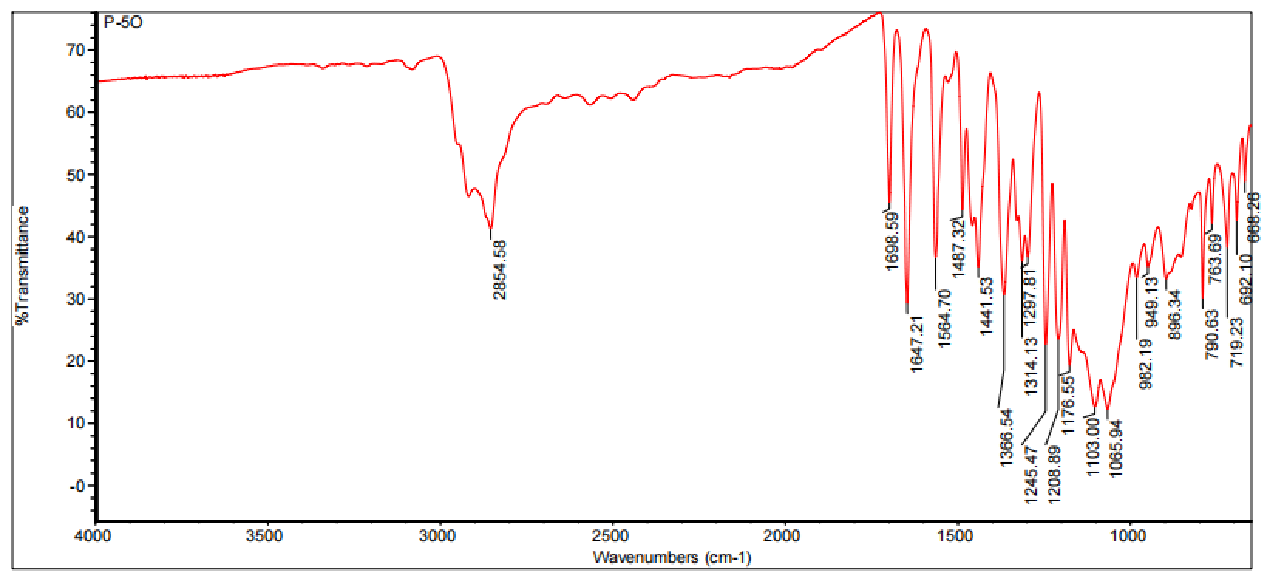


**Figure S9**. IR spectra of P-4O and P-5O.


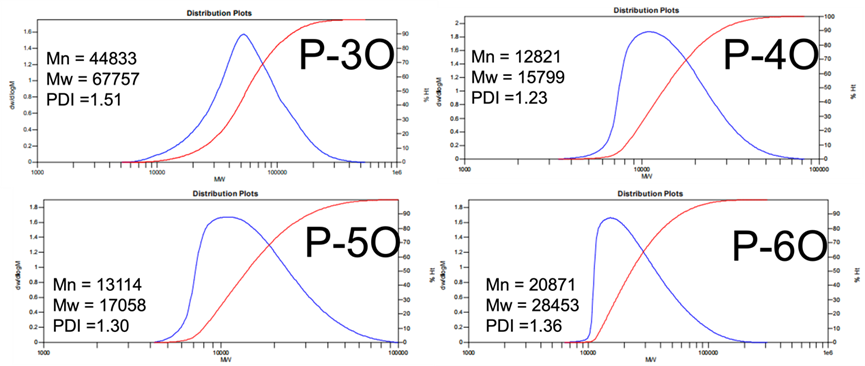


**Figure S10**. GPC plots of NDI-2Tz based conjugated polymer P-3O, P-4O, P-5O and P-6O.

**2. Thermal Properties**


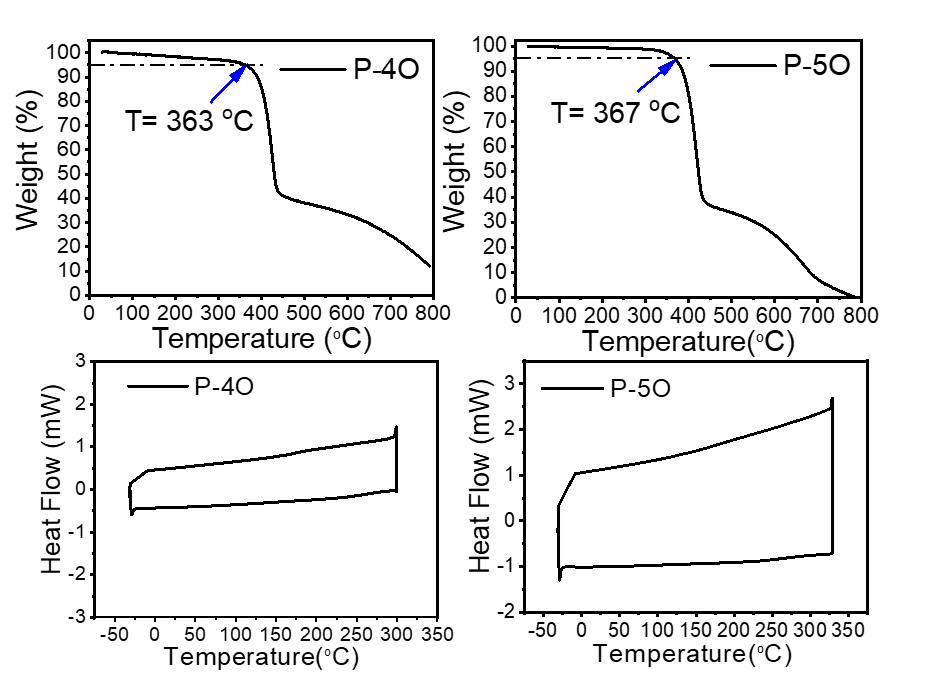


**Figure S11.** Thermogravimetric analysis plots for P-4O and P-5O. and DSC analysis plots for P-4O and P-5O on second heating, 10 °C /min.

**3. Absorption Profiles Characterizations**


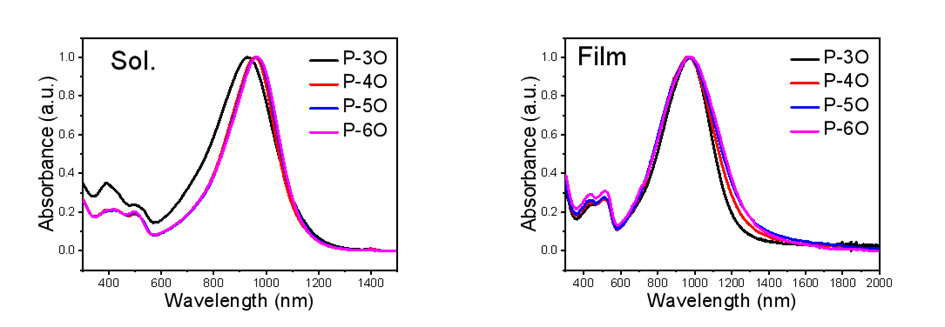


**Figure S12.** The UV-VIS-NIR absorption of P-3O, P-4O, P-5O and P-6O in dilute CHCl_3_ (10^-5^ M) and thin film state.

**4. Output curves of the NDI-2Tz copolymers-based OECT**

**
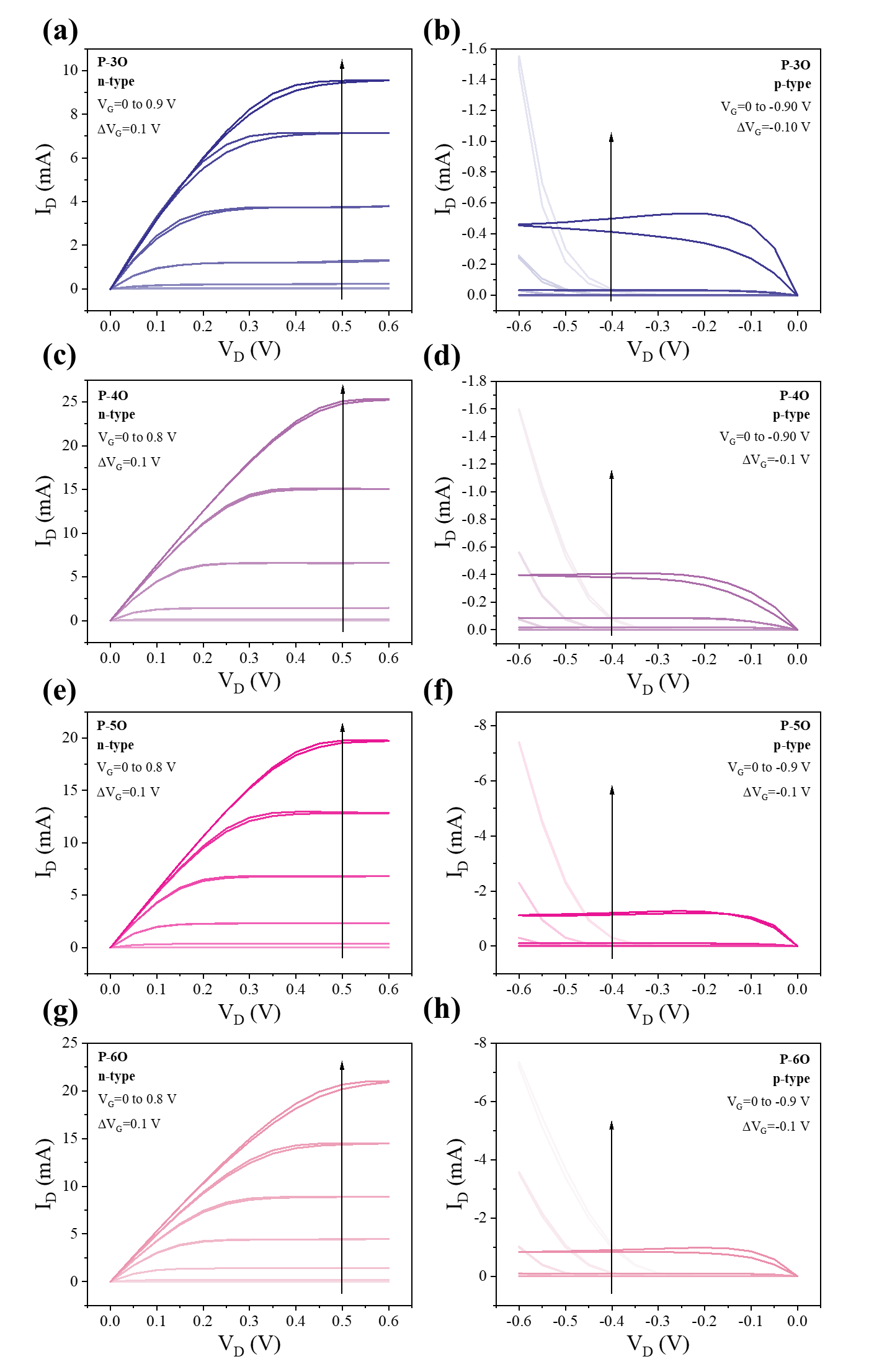
**

**Figure S13.** The output curves of the NDI-2Tz copolymers-based OECT. (a-b) P-3O, (c-d) P-4O, (e-f) P-5O, (g-h) P-6O.

**5. Water Contact Angle**

The hydrophilicity of P3O-P6O was evaluated by water contact angle measurements using a contact angle goniometer (CA100, Guangdong Beidou Precision Instrument Co., Ltd.). Prior to the test, polymer solution (5 mg/ml in chloroform) was spin-coated onto the ITO glass, followed by annealing at 100 ℃ for 30 min. The average water contact angle value was calculated based on measurements from at least 5 locations for each sample.


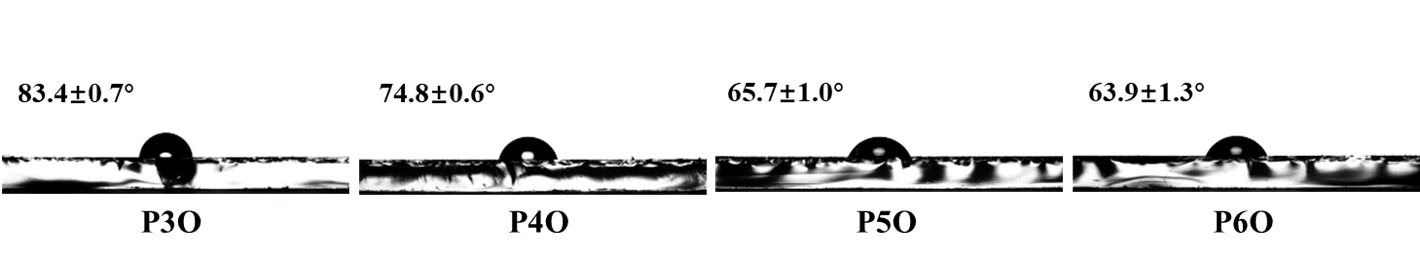


**Figure S14.** Images for water contact angle of P-3O, P-4O, P-5O and P-6O.

**6. AFM measurement of the NDI-2Tz polymers**


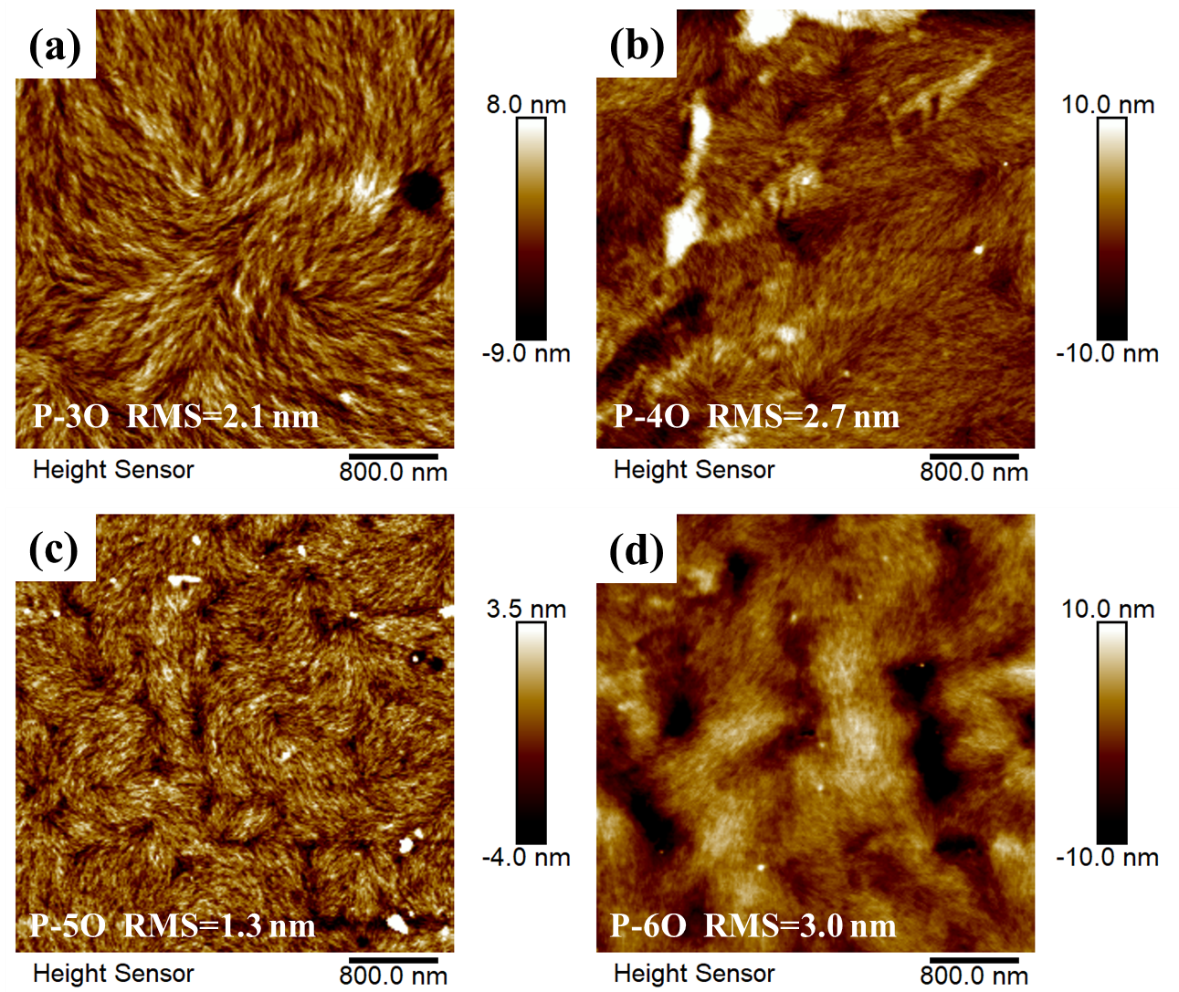


**Figure S15.** AFM height images (scale bar: 4 μm) of (a) P-3O, (b) P-4O, (c) P-5O, (d) P-6O.

**7. Calculation of the μC* value for NDI-2Tz copolymers**

**Figure S16.** Plots of transconductance gm versus channel geometry and operating parameters of P-3O, P-4O, P-5O and P-6O from which [μC*] values were extracted.

**8. Operation Stability**


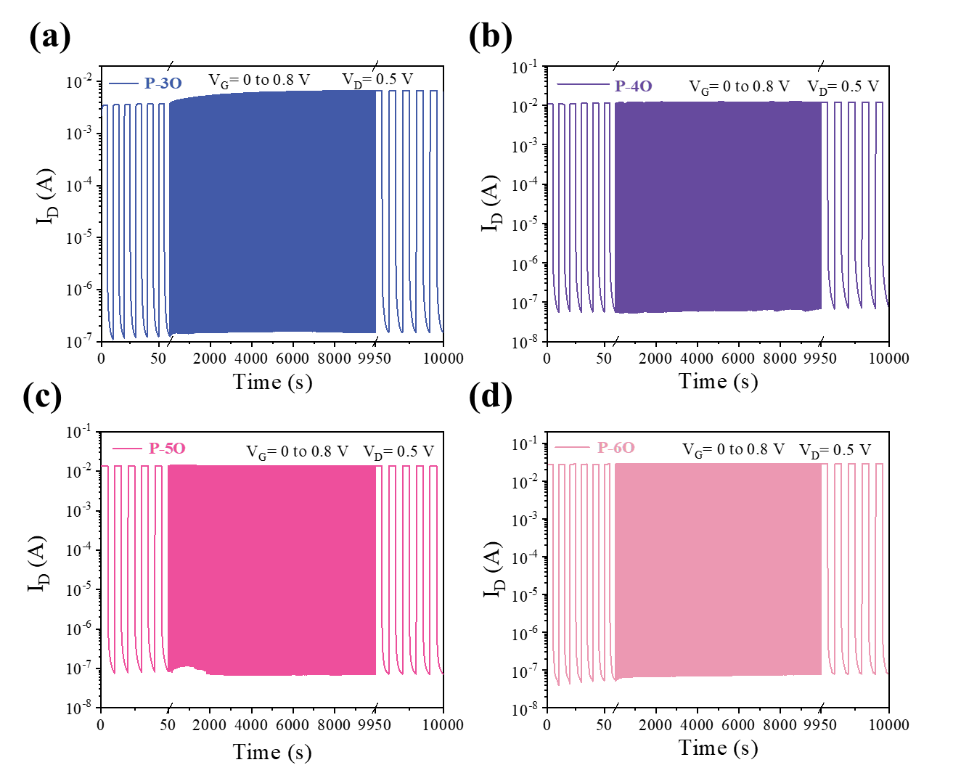


**Figure S17.** OECTs operation stability measurements were conducted for different polymers. (a) P-3O, (b) P-4O. (c) P-5O, and (d) P-6O. Drain currents (I_ds_) were monitored for 1000 pulse cycles during which a gate voltage pulse *V_G_* = 0.8 V was applied for 5 s with an interval time of 5 s (*V_D_* = 0.5 V).

**9. The characteristics of P-4O-based OECT.**


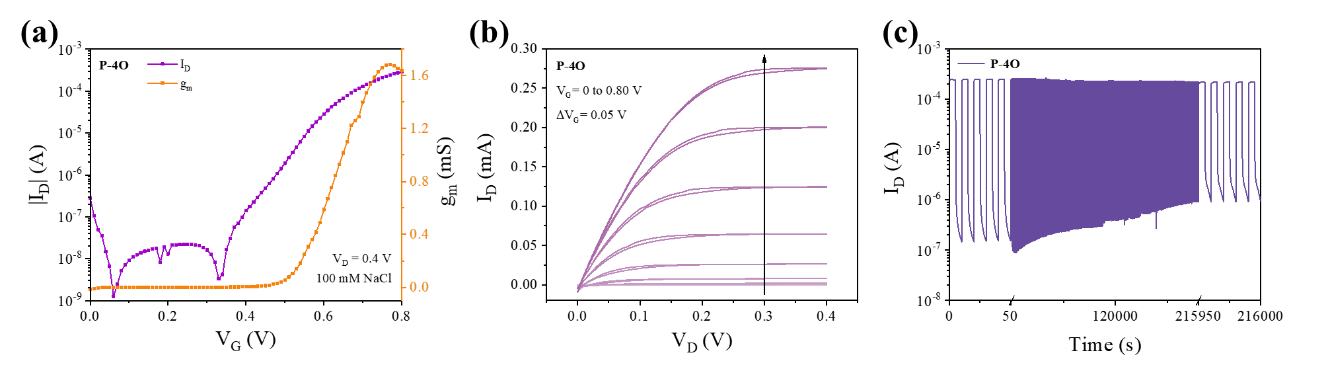


**Figure S18**. (a) Transfer characteristic, (b) output characteristic, and (c) stability of P-4O-based OECT devices with channel dimensions of W/L= 3000 / 200 μm.

**10. Voltage gain of the inverter at various supply voltages.**

Figure S19-1 presents the results obtained under the testing conditions where V_in_ was scanned from 0 V to 0.8 V and then back to 0 V. Notably, an overshoot occurred during the initial testing stage due to sudden voltage input. Similarly, we conducted a test where V_in_ was scanned from 0.8 V to 0 V and then back to 0.8 V (as shown in Figure S19-2) and observed an overshoot phenomenon near the initial 0.8 V. Therefore, we believe this is attributed to the testing equipment rather than the device itself. In the main manuscript, the results presented are based on the data from Figure S19-1 after excluding the first 200 data points.


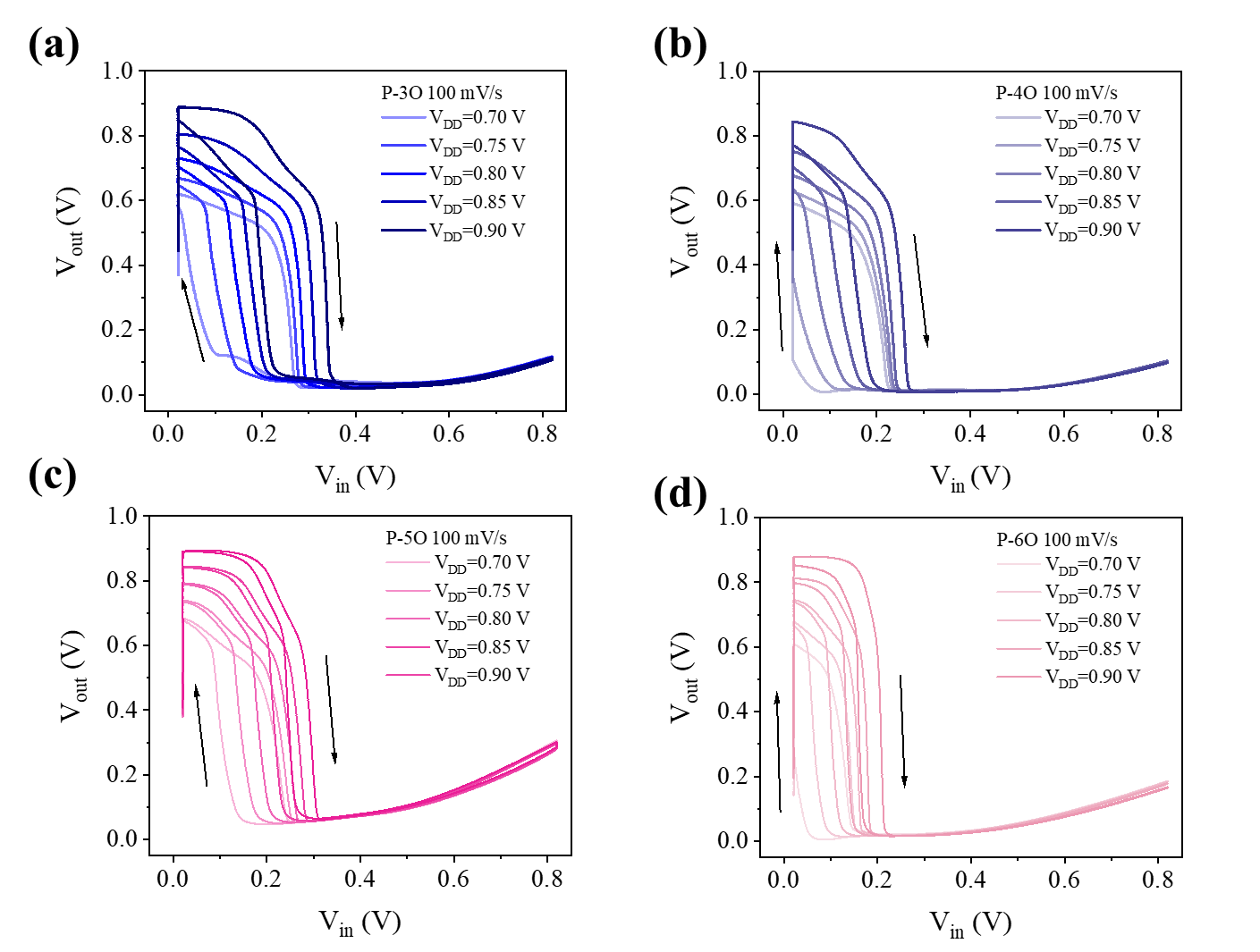


**Figure S19-1.** Initial inverter characteristic curves, V_in_ is scanned from 0 V to 0.8 V and then back to 0 V. (a) P-3O, (b) P-4O, (c) P-5O, (d) P-6O.


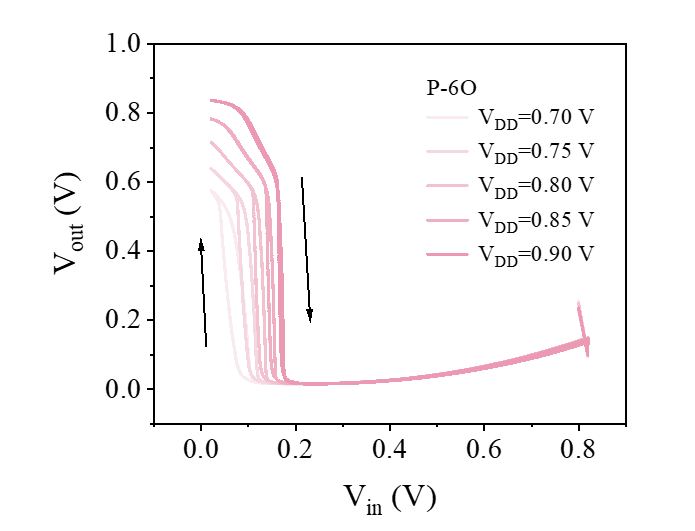


**Figure S19-2.** Initial inverter characteristic curve of P-6O. V_in_ is scanned from 0.8 V to 0 V and then back to 0.8 V.


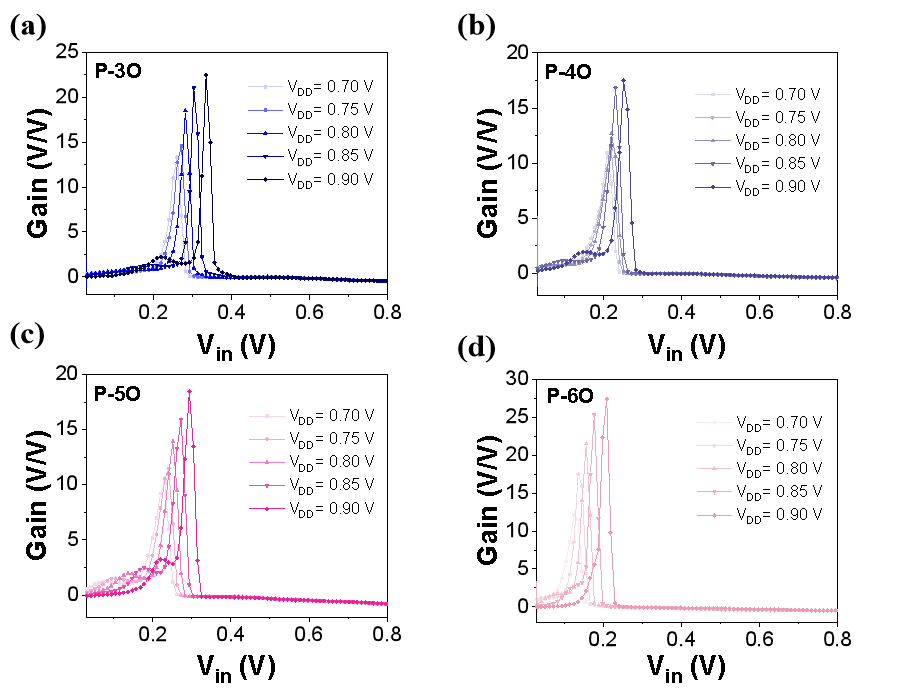


**Figure S20.** The voltage gain of the inverter at various supply voltages. (a) P-3O, (b) P-4O, (c) P-5O, (d) P-6O.

**11. CV in Aqueous Electrolyte (100 mM NaCl)**


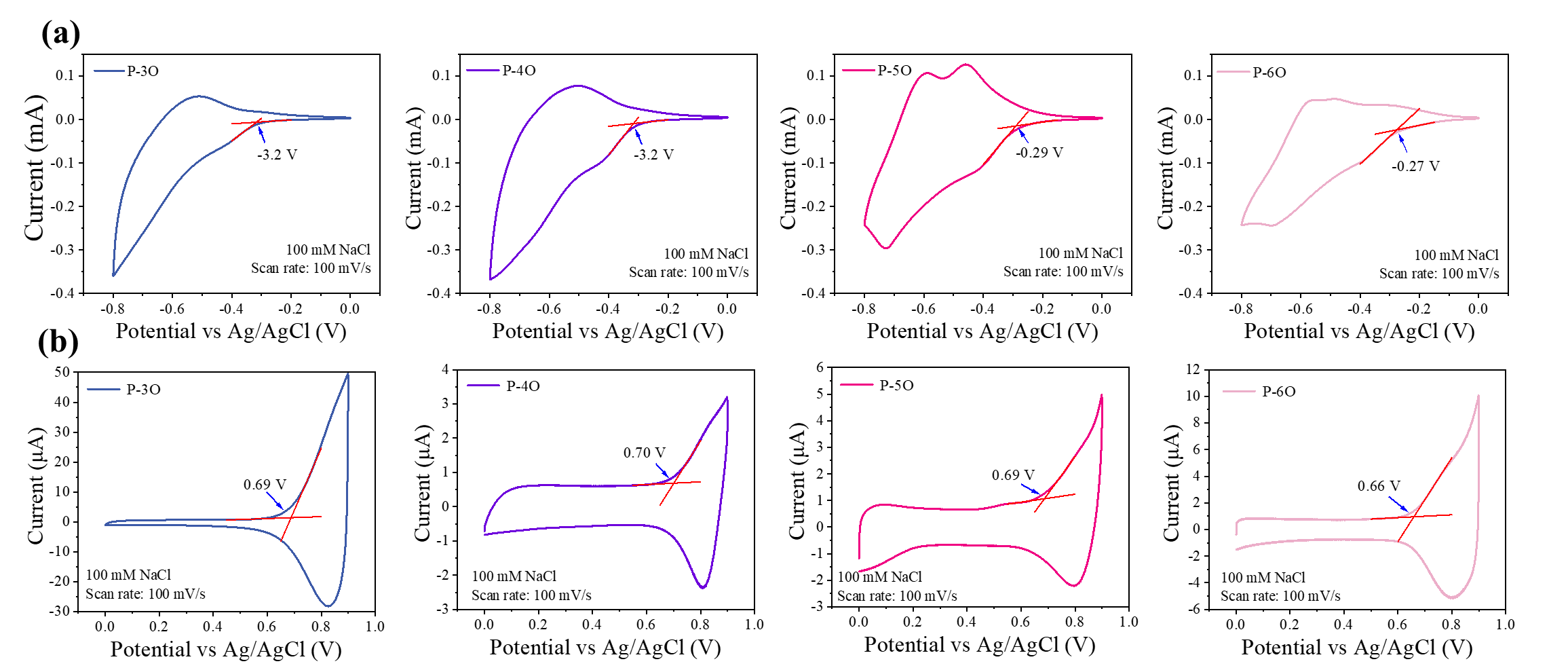


**Figure S21.** Cyclic voltammetry of polymer thin films (P-3O, P-4O, P-5O, P-6O) on ITO substrates in 100 mM NaCl aqueous solution with both (a) negative potential (reduction, n-type doping) and (b) positive potential (oxidation, p-type doping).

**12. Electrochemical Spectroscopy**


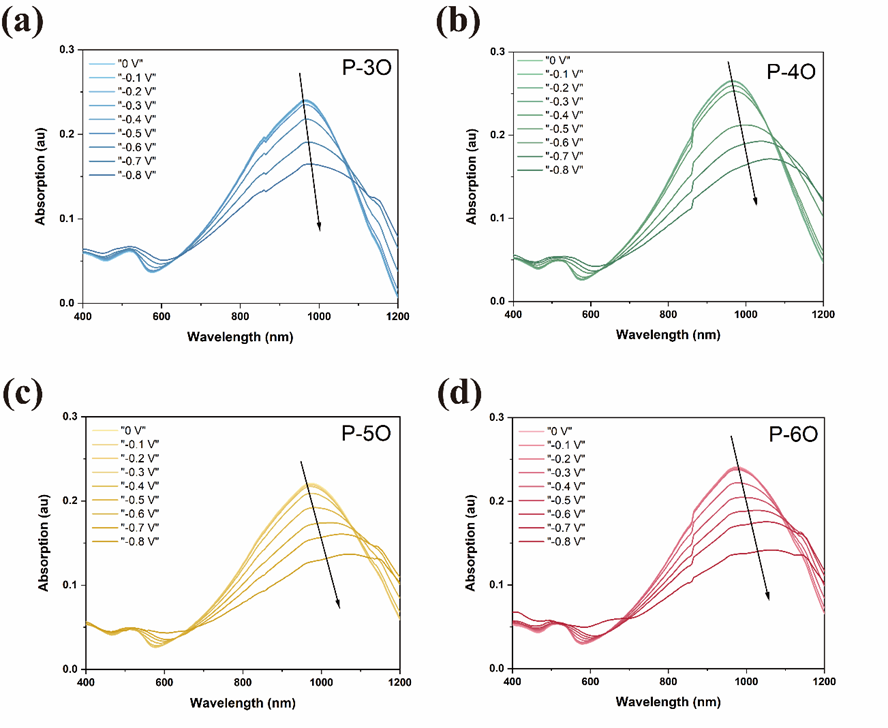


**Figure S22.** Electrochemical spectroscopy of (a) P-3O, (b) P-4O, (c) P-5O, and (d) P-6O with the negative potential versus Ag/AgCl in 100 mM NaCl aqueous solution (reduction, n-type doping).

**13. Density Functional Theory Calculation**

DFT calculations were performed using the Gaussian 09 program with B3LYP functional and 6-311G** basis set. One repeat unit of each polymer as the model molecule was applied in our simulation to reduce the calculation time. These model molecules were first optimized in the gas phase. Then, we used the optimized geometry as the starting point for further single-point energy calculations.


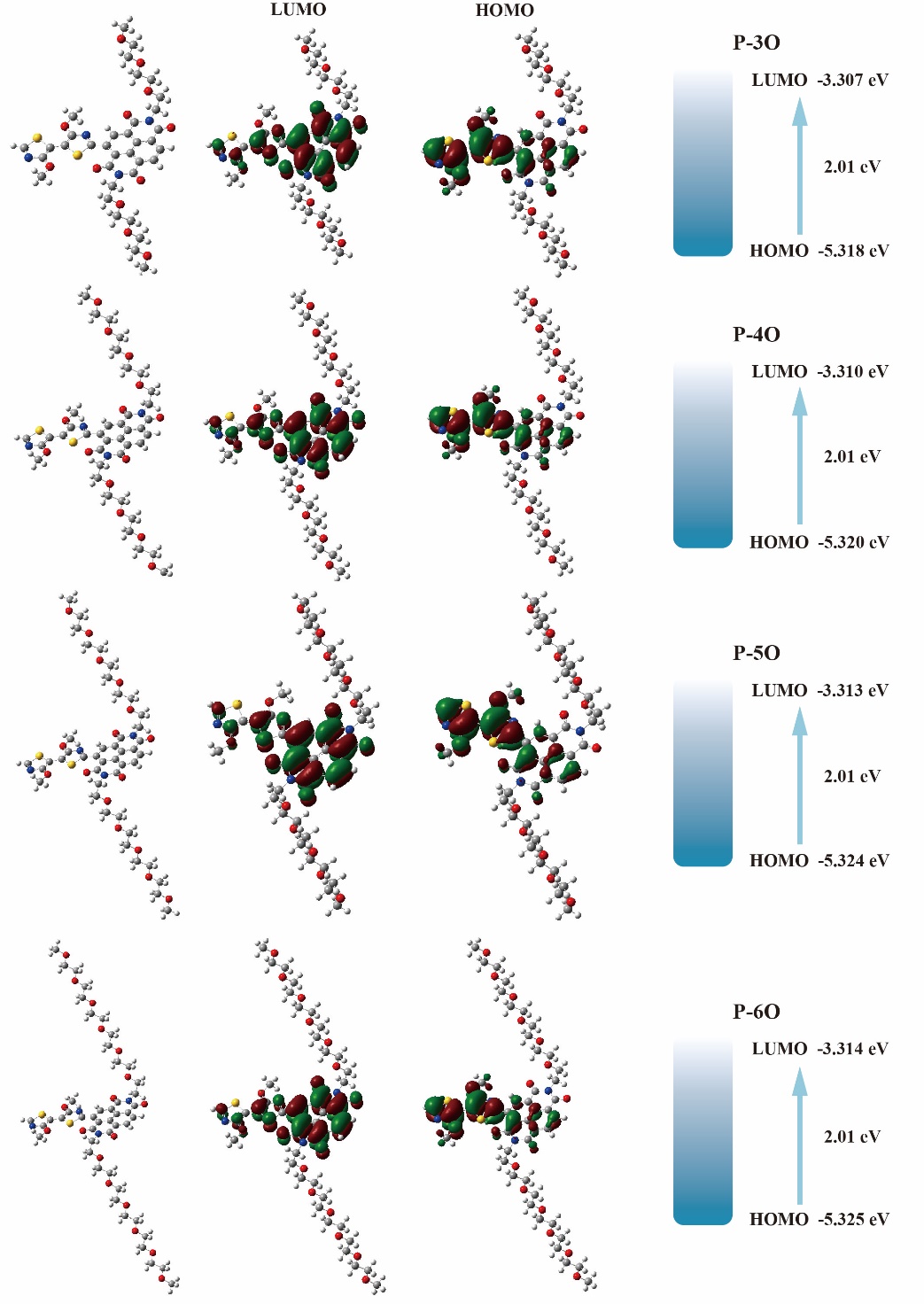


**Figure S23.** Optimized molecular geometries for one repeat unit of P-3O, P-4O, P-5O, and P-6O. The calculation was performed at the B3LYP/6-31G(d,p) level.

**14. Cyclic Voltammetry (CV)**


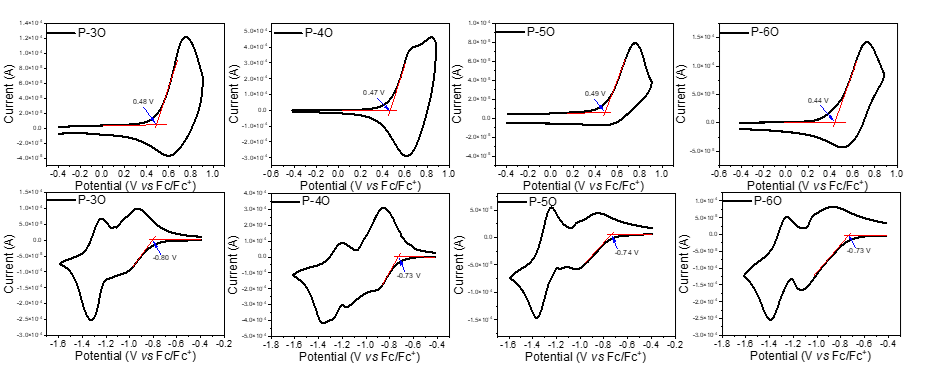


**Figure S24.** Cyclic voltammograms of the conjugated polymer P-3O, P-4O, P-5O, and P-6O thin films deposited on the glassy carbon working electrode in CHCN_3_ solution containing Bu_4_NPF_6_ electrolyte.

**15. GIWAXS**


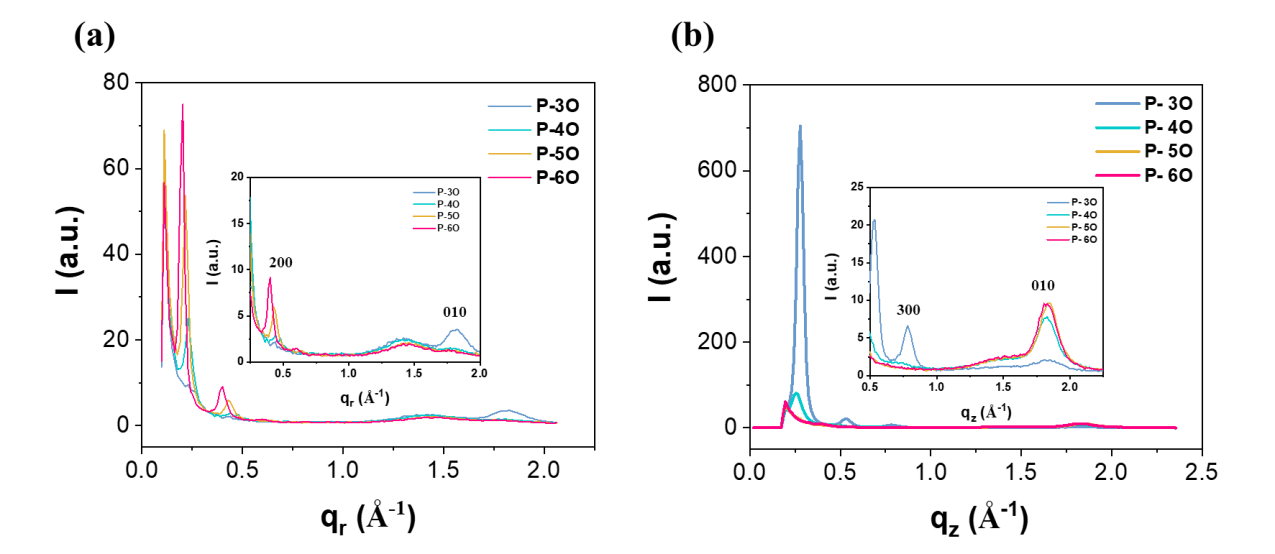


**Figure S25.** The GIWAXS (a) horizontal (in-plane) and (b) vertical (out-of-plane) line cuts for P-3O, P-4O, P-5O, and P-6O thin films.

**Table S1.** Solid states packing parameters of P-3O、P-4O、P-5O and P-6O in the out-of-plane (OOP) and in-plane (IP) direction.

| π-π stack  010 |  | P-3O  (IP) | P-4O (OOP) | P-5O (OOP) | P-6O (OOP) |
| --- | --- | --- | --- | --- | --- |
|  | q (Å^-1^) | 1.81 | 1.82 | 1.84 | 1.82 |
|  | d-spacing (Å) | 3.47 | 3.45 | 3.41 | 3.45 |
|  | FWHM (Å^-1^) | 0.137 | 0.142 | 0.146 | 0.15 |
|  | Correlation length (Å) | 40.82 | 39.38 | 38.30 | 37.28 |
|  | g | 0.110 | 0.111 | 0.112 | 0.114 |

**Reference**

[1] D. Komáromy, M. C. A. Stuart, G. Monreal Santiago, M. Tezcan, V. V. Krasnikov, S. Otto, *J. Am. Chem. Soc.* **2017**, 139, 6234.

[2] H. F. Higginbotham, S. Maniam, S. J. Langford, T. D. M. Bell, *Dyes and Pigments* **2015**, 112, 290.

[3] A. Alliband, F. A. Meece, C. Jayasinghe, D. H. Burns, *J. Org. Chem.* **2013**, 78, 356.

[4] Y. Zhang, E. R. W. van Doremaele, G. Ye, T. Stevens, J. Song, R. C. Chiechi, Y. van de Burgt, *Adv. Mater.* **2022**, 34, 2200393.

[5] J. Liu, G. Ye, H. G. O. Potgieser, M. Koopmans, S. Sami, M. I. Nugraha, D. R. Villalva, H. Sun, J. Dong, X. Yang, X. Qiu, C. Yao, G. Portale, S. Fabiano, T. D. Anthopoulos, D. Baran, R. W. A. Havenith, R. C. Chiechi, L. J. A. Koster, *Adv. Mater.* **2020**, 33, 2006694.
